# Assessment of folate receptor-β expression in human neoplastic tissues

**DOI:** 10.18632/oncotarget.3739

**Published:** 2015-03-30

**Authors:** Jiayin Shen, Karson S. Putt, Daniel W. Visscher, Linda Murphy, Cynthia Cohen, Sunil Singhal, George Sandusky, Yang Feng, Dimiter S. Dimitrov, Philip S. Low

**Affiliations:** ^1^ Department of Chemistry, Purdue University, West Lafayette, IN, USA; ^2^ Center for Drug Discovery, Purdue University, West Lafayette, IN, USA; ^3^ Department of Laboratory Medicine and Pathology, Mayo Clinic, Rochester, MN, USA; ^4^ Department of Biochemistry and Molecular Biology, Mayo Clinic College of Medicine, Rochester, MN, USA; ^5^ Department of Pathology and Laboratory Medicine, Emory University Hospital, Atlanta, GA, USA; ^6^ Division of Thoracic Surgery, Department of Surgery, Hospital of the University of Pennsylvania School of Medicine, Philadelphia, PA, USA; ^7^ Department of Pathology, Indiana University School of Medicine, Indianapolis, IN, USA; ^8^ Protein Interactions Section, Laboratory of Experimental Immunology, Cancer and Inflammation Program, Center for Cancer, National Cancer Institute-Frederick, National Institutes of Health, Frederick, MD, USA

**Keywords:** folate receptor, folate receptor beta, activated macrophage, tumor associated macrophage

## Abstract

Over-expression of folate receptor alpha on cancer cells has been frequently exploited for delivery of folate-targeted imaging and therapeutic agents to tumors. Because limited information exists on expression of the beta isoform of the folate receptor in human cancers (FR-β), we have evaluated the immunohistochemical staining pattern of FR-β in 992 tumor sections from 20 different human cancer types using a new anti-human FR-β monoclonal antibody. FR-β expression was shown to be more pronounced in cells within the stroma, primarily macrophages and macrophage-like cells than cancer cells in every cancer type studied. Moreover, FR-β expression in both cancer and stromal cells was found to be statistically more prominent in females than males. A significant positive correlation was also observed between FR-β expression on stromal cells and both the stage of the cancer and the presence of lymph node metastases. Based on these data we conclude FR-β may constitute a good target for specific delivery of therapeutic agents to activated macrophages and that accumulation of FR-β positive macrophages in the stroma could serve as a useful indicator of a tumor's metastatic potential.

## INTRODUCTION

Folic acid (vitamin B9) is required for one-carbon metabolism, which is involved in *de novo* synthesis of nucleotides, methylation of DNA, carboxymethylation of G proteins and synthesis of many important metabolic intermediates [1]. Folic acid is taken into cells via the reduced folate carrier [2], the proton coupled folate transporter [3] or one of four isoforms (α, β, γ, δ) of the folate receptor (FR) [4]. Although FR's 10^4^-fold higher affinity for folate (*K*_d_ ~ 10^−10^ M) may render the receptor the preferred pathway for uptake of the vitamin when folate concentrations are low, the restricted expression of FR and its general inaccessibility in normal tissues may limit this potential benefit to very few cells [5]. Thus, FR-α is expressed primarily on the apical surfaces of epithelial cells lining the openings in the lungs, kidneys, mammary ducts and choroid plexus, where it only encounters folates within the respective lumens [6, 7, 8]. Similarly, FR-β has only been reported on activated, but not resting or quiescent myeloid cells (primarily monocytes and macrophages) [9, 10, 11], which constitute only a small fraction of the total myeloid cell population. FR-γ may be secreted in very low quantities into the bloodstream where it is difficult to detect [12, 13], and FR-δ has only been observed on regulatory T cells and ova [14].

In contrast to its limited expression in normal tissues, FR-α has been found in many types of cancer, including cancers of the ovary, lung, kidney, breast, colon and endometrium [15]. Indeed, up to 40% of human cancers have been recently estimated to over-express FR-α [15]. Thus, FR-α has not only become an established tumor cell marker, but it has also been exploited for the selective delivery of both imaging and therapeutic agents to each of the above malignancies [16, 17, 18]. Not surprisingly, six folate-targeted drugs are currently undergoing human clinical trials [19, 20, 21, 22], and others are in various stages of preclinical development. One folate-vinca alkaloid conjugate (EC145) has even advanced to phase III clinical trials [22] in combination with its companion diagnostic agent, folate-^99m^Tc (EC20) [20].

Largely due to the absence of a commercial monoclonal antibody for FR-β, little information exists on the expression of FR-β in malignant tissues. While the β isoform of the folate receptor has recently been shown to be expressed on tumor-associated macrophages [9, 10, 23], the only reports of FR-β expression on malignant cells reveal its presence in myelogenous leukemias [24]; i.e. consistent with its expression on the corresponding nontransformed cells. In an effort to ascertain the expression pattern of FR-β in nonmyelogenous malignancies, we have exploited a recently developed monoclonal antibody to human FR-β [25] to stain nearly 1000 human tumor tissue sections from 20 different tumor types. We report here that FR-β positive tumor associated macrophage-like cells are present in most human cancers and that several human malignancies that derive from FR-β negative cells also express the β isoform of FR.

## RESULTS AND DISCUSSION

### Characterization of the biotinylated-m909 antibody

**Figure 1:**
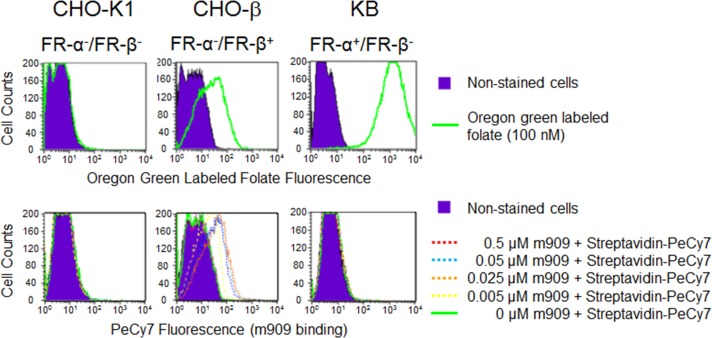
Characterization of biotin-labeled m909 on cultured cells Mock CHO-K1 cells, human FR-β stably-transfected CHO cells (CHO-β) or KB cells that naturally express FR-α were incubated with Oregon green labeled folate (100 nM) or serial concentrations of biotin-m909 followed by Streptavidin-PeCy7 for quantitation. Fluorescent intensity of individual cells was determined by flow cytometry.

The specificity of the anti-human FR-β human monoclonal antibody m909 has been previously established by ELISA, surface plasmon resonance, flow cytometry, confocal microscopy and immunohistochemistry (IHC) staining in a small tissue microarray [11, 23, 25], however its use for IHC staining of large tumor microarrays has not been reported. After exploring multiple staining protocols, it was found that use of biotin-derivatized m909 in combination with streptavidin-HRP staining yielded the highest resolution images. To ensure that attachment of biotin to m909 did not compromise selectivity of the antibody for FR-β, binding of biotinylated-m909 was compared with CHO-K1 cells that do not express either FR-α or FR-β [26], CHO-K1 cells transfected with a stable human FR-β expression vector (herein referred to as CHO-β) and KB cells that naturally express high levels of FR-α, but not FR-β [27]. For this purpose, all three cell lines were incubated with Oregon green labeled folate and analyzed via flow cytometry to test for expression of a functional folate receptor. As expected, mock infected CHO-K1 cells did not display any folate-Oregon green binding, while both the CHO-β and KB cells showed large shifts in fluorescence, indicating binding of the fluorescent folate (Figure 1). When these same cell lines were incubated with various concentrations of biotinylated-m909 followed by PeCy7-labeled Streptavidin, the labeled CHO-K1 and the FR-α expressing KB cells were indistinguishable from the non-labeled cells, indicating no binding of the biotinylated-m909 antibody (Figure 1). In contrast, a dose-dependent change in fluorescence intensity was observed when biotinylated m909 was incubated with FR-β expressing CHO-β cells (Figure 1). Taken together, these data indicate that biotinylation of m909 has little effect on FR-β selectivity.

### Biotinylated-m909 immunohistochemistry in neoplastic human tissues

**Figure 2:**
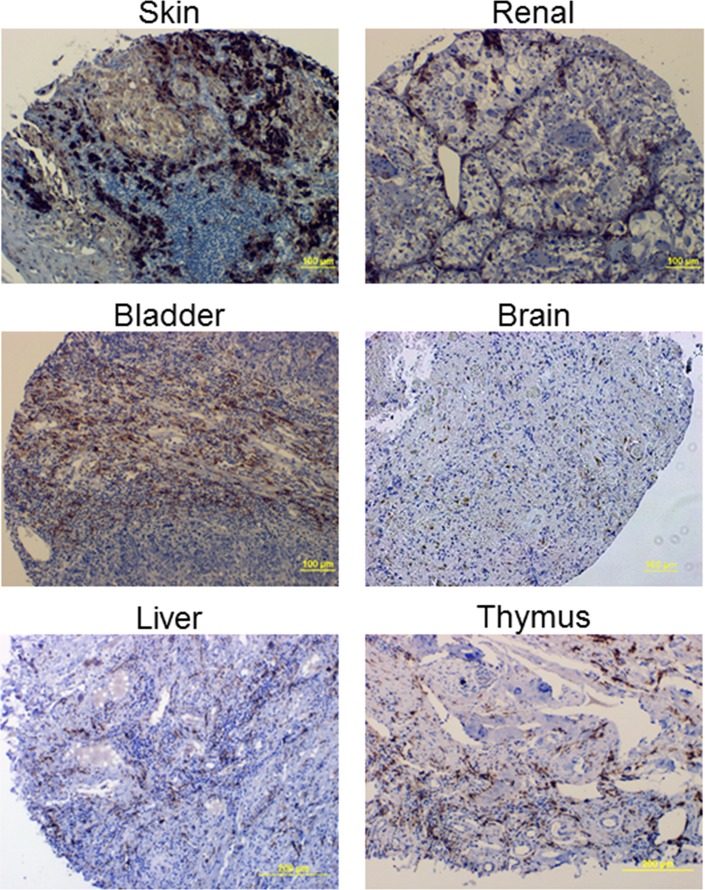
Example IHC images using the FR-β specific m909 antibody in various cancer tissues

To determine FR-β expression in both malignant and nonmalignant cells of multiple human cancers, IHC was performed with the biotinylated m909 antibody on human tumor tissue microarrays from a variety of providers. Several representative images are shown in Figure 2 from different cancer tissue types. Evaluation of the staining in both cancer and stromal cells was performed by trained pathologists, and in total 992 tumor specimens were analyzed.

**Figure 3:**
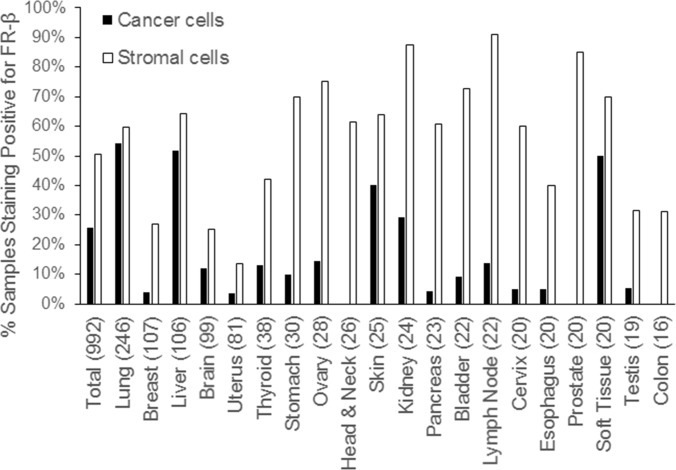
Prevalence of FR-β expressing cancer and stromal cells in various human tumor sections The x-axis shows the tissue of origin of the cancer with the number of samples tested in parenthesis.

As shown in Table 1 and Figure 3, FR-β expression on cancer cells was observed in nearly one quarter of all samples, with prominent staining noted primarily in malignancies of the lung, liver, skin, kidney and soft tissue. No FR-β expression was observed in malignant cells of head & neck, prostate and colon cancers. In contrast, FR-β expression was evident on stromal cells in slightly over half of the cancer sections examined. Where closely evaluated by trained pathologists, the FR-β positive stromal cells appeared to be macrophages or macrophage-like cells. Previous studies using cancer and inflamed tissues have shown that FR-β positive cells are predominantly monocytes/macrophages [9, 10, 11, 23, 28, 29].

**Table 1:** Prevalence of FR-β expressing cancer and stromal cells in various human tumor sections

Tissue Type (n)	Tumor Type (n)	Sections staining Cancer Cells		positive for FR-β Stromal Cells	
		n	%	n	%
**Total (992)**		**255**	**26%**	**503**	**51%**
**Lung (246)**		**133**	**54%**	**147**	**60%**
	adenocarcinoma (230)	129	56%	138	60%
	squamous cell carcinoma (14)	2	14%	7	50%
	non-small cell carcinoma (2)	2	100%	2	100%
**Breast (107)**		**4**	**4%**	**29**	**27%**
	invasive ductal carcinoma (78)	2	3%	22	28%
	infiltrating ductal carcinoma (10)	2	20%	4	40%
	medulary carcinoma (4)	0	0%	1	25%
	mutinous carcinoma (4)	0	0%	0	0%
	intraductal papillary carcinoma (3)	0	0%	0	0%
	intraductal carcinoma (2)	0	0%	0	0%
	invasive cribriform carcinoma (2)	0	0%	0	0%
	invasive lobular carcinoma (1)	0	0%	1	100%
	invasive papillary carcinoma (1)	0	0%	0	0%
	metaplastic carcinoma (1)	0	0%	1	100%
	mixed type invasive lobular cacinoma (1)	0	0%	0	0%
**Liver (106)**		**55**	**52%**	**68**	**64%**
	hepatocellular carcinoma (104)	55	53%	68	65%
	mucinous adenocarcinoma (2)	0	0%	0	0%
**Brain (99)**		**12**	**12%**	**25**	**25%**
	glioblastoma (49)	9	18%	16	33%
	astrocytoma (30)	3	10%	4	13%
	oligo-dendroglioma (6)	0	0%	3	50%
	anaplatic oligodendroglioma (3)	0	0%	1	33%
	medulloblastoma (3)	0	0%	0	0%
	oligo-astrocytoma (3)	0	0%	0	0%
	ependymoma (2)	0	0%	0	0%
	anaplastic ependymoma (1)	0	0%	0	0%
	malignant ependymoma (1)	0	0%	1	100%
	malignant oligodendroglioma (1)	0	0%	0	0%
**Uterus (81)**		**3**	**4%**	**11**	**14%**
	endometrial adenocarcinoma (43)	2	5%	11	26%
	endometrial simple hyperplasia (10)	0	0%	0	0%
	metastatic endometrial adenocarcinoma (5)	0	0%	0	0%
	squamous cell carcinoma (5)	1	20%	0	0%
	chronic endometritis (4)	0	0%	0	0%
	endometrial glandular cystic hyperplasia (4)	0	0%	0	0%
	Enometrial polyp (3)	0	0%	0	0%
	hyperplasia - smooth muscle tissue (2)	0	0%	0	0%
	acute enometritis (1)	0	0%	0	0%
	endometrial adenomatous hyperplasia (1)	0	0%	0	0%
	hyperplasia of endometrium - sparse (1)	0	0%	0	0%
	moderate atypical hyperplasia of endometrium (1)	0	0%	0	0%
	severe atypical hyperplasia of endometrium (1)	0	0%	0	0%
**Thyroid (38)**		**6**	**13%**	**16**	**42%**
	thymoma (19)	0	0%	0	0%
	papillary carcinoma (10)	3	30%	9	90%
	follicular carcinoma (9)	2	22%	7	78%
**Stomach (30)**		**3**	**10%**	**21**	**70%**
	adenocarcinoma (27)	3	11%	18	67%
	signet ring cell carcinoma (2)	0	0%	2	100%
	mucinous adenocarcinoma (1)	0	0%	1	100%
**Ovary (28)**		**4**	**14%**	**21**	**75%**
	papillary serous cystadenocatinoma (12)	1	8%	7	58%
	mucinous adenocarcinoma (7)	1	14%	5	71%
	serous carcinoma (4)	2	50%	4	100%
	adenocarcinoma (2)	0	0%	2	100%
	papillary serous adenocarcinoma (3)	0	0%	3	100%
**Head & Neck (26)**		**0**	**0%**	**16**	**62%**
	squamous cell carcinoma (26)	0	0%	16	62%
**Skin (25)**		**10**	**40%**	**16**	**64%**
	malignant melanoma (25)	10	40%	16	64%
**Kidney (24)**		**7**	**29%**	**21**	**88%**
	clear cel carcinoma (20)	3	15%	17	85%
	renal cel carcinoma (4)	4	100%	4	100%
**Pancreas (23)**		**1**	**4%**	**14**	**61%**
	adenocarcinoma (22)	1	5%	14	64%
	acinic cell carcinoma (1)	0	0%	0	0%
**Bladder (22)**		**2**	**9%**	**16**	**73%**
	transitional cell carcinoma (22)	2	9%	16	73%
**Lymph Node (22)**		**3**	**14%**	**20**	**91%**
	hodgkin's disease (9)	0	0%	9	100%
	diffuse B cell lymphoma (8)	0	0%	7	88%
	metastatic neoplasm (4)	3	75%	3	75%
	anaplasbc large cell lymphoma (1)	0	0%	1	100%
**Cervix (20)**		**1**	**5%**	**12**	**60%**
	squamous cell carcinoma (20)	1	5%	12	60%
**Esophagus (20)**		**1**	**5%**	**8**	**40%**
	adenocarcinoma (10)	0	0%	2	20%
	squamous cell carcinoma (10)	1	10%	6	60%
**Prostate (20)**		**0**	**0%**	**17**	**85%**
	adenocacinoma (20)	0	0%	17	85%
**Soft Tissue (20)**		**10**	**50%**	**14**	**70%**
	fibrosarcoma (10)	7	70%	5	50%
	liposarcoma (5)	1	20%	4	80%
	mucinous liposarcoma (4)	1	25%	4	100%
	spindle cell type liposarcoma (1)	1	100%	1	100%
**Testis (19)**		**1**	**5%**	**6**	**32%**
	seminoma (15)	1	7%	4	27%
	embryonal carcinoma (4)	0	0%	2	50%
**Colon (16)**		**0**	**0%**	**5**	**31%**
	adenocacinoma (16)	0	0%	5	31%

### FR-β staining intensity and patient correlations

To explore possible correlates of FR-β expression, the staining intensity (a crude measure of receptor concentration) of a multi-cancer TMA from BioMax was evaluated by a trained pathologist and scored on a scale of 0 to 3. As shown in SI Figure 1, highly significant difference was apparent in the staining intensities of cancer cells versus stromal cells, with stromal cells showing a nearly 8-fold greater average intensity than the cancer cells (*p*-value = 1.54 × 10^−57^). This trend of greater staining intensity occurred in nearly all cancers tested except malignancies of the skin and soft tissue, where the staining intensities were nearly equal (SI Figure 2). Since FR expression is highly tissue specific and because most normal tissues do not express FR [30], the high levels of FR-β found in stromal cells of tumors may at first seem unusual. However, recent observations that tumor associated macrophages express FR-β [9, 10, 23] and that many tumors accumulate these activated macrophages [9, 10, 23] argue that this result should have been anticipated.

**Figure 4:**
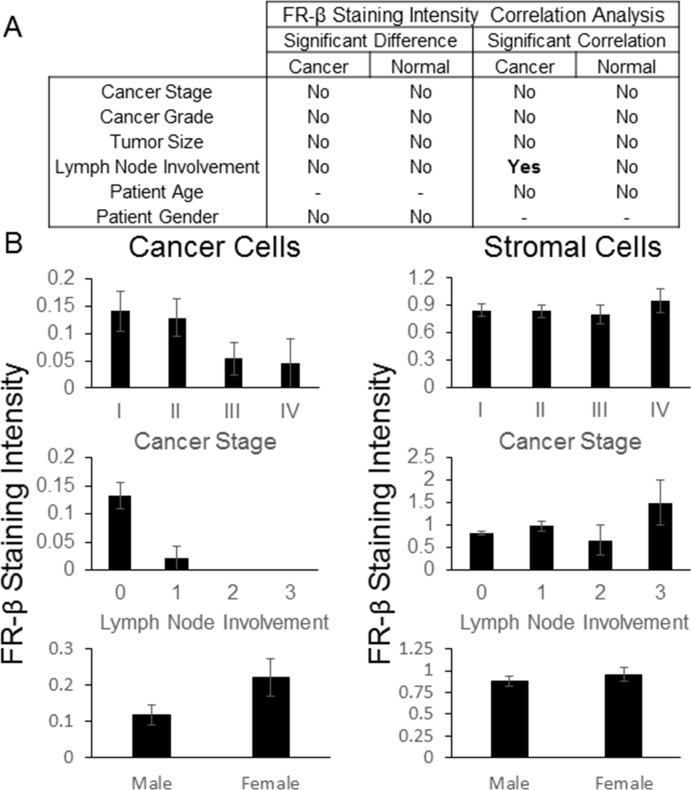
FR-β staining intensity correlations IHC was performed on a BioMax multi-cancer tissue microarray using the FR-β specific monoclonal antibody m909. The relative staining intensity, graded on a scale of 0 to 3, of positively staining cells within the tumor and stroma were determined. **A**) Statistically significant differences (*p*-value <0.05) using ANOVA or a t-test and correlations using a Spearman correlation (*p*-value <0.05) between the average FR-β staining intensity and various pathological data are summarized. **B**) Graphs for select FR-β staining intensity and various pathological data (error bars represent SEM) are shown. Lymph node involvement was based upon the AJCC/UICC stage - TxNxMx. Additional graphs and data can be found in the Supporting Information.

Next, the derived staining rankings were examined for any relationships with patient pathology data, where available, using a t-test, 1-way ANOVA or Spearman correlation to determine significance (Figure 4 and SI Figures 1-8). As summarized in Figure 4A and 4B, no correlations were found between stromal cell staining intensity and available patient data. However, for cancer cells, a weakly significant correlation was noted between staining intensity and lymph node involvement (*p*-value = 0.0323). A general inverse correlation between cancer stage and FR-β expression on malignant cells was also observed (Figure 4B), but this relationship was not statistically significant (*p*-value = 0.1041).

### FR-β staining percentage and patient correlations

**Figure 5:**
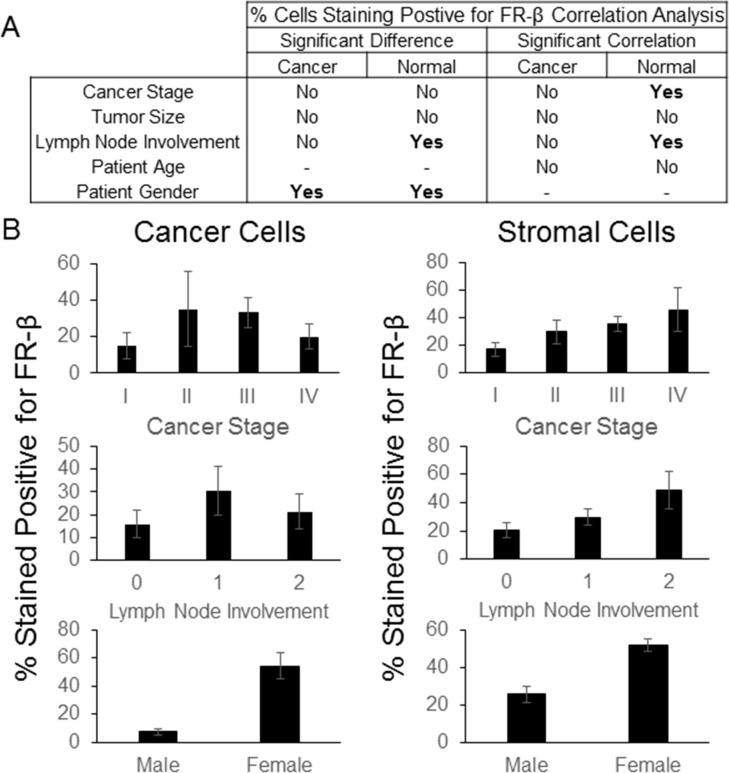
FR-β staining correlations IHC was performed on a Asterand tissue microarray using the FR-β specific monoclonal antibody m909. The approximate percentage of positively staining cells within the tumor and stroma were determined. **A**) Statistically significant differences (*p*-value <0.05) using ANOVA or a t-test and correlations (*p*-value <0.05) using a Spearman correlation between the percentage of FR-β staining cells and various pathological data are summarized. **B**) Graphs for select FR-β staining and various pathological data (error bars represent SEM) are shown. Lymph node involvement was based upon the AJCC/UICC stage - TxNxMx. Additional graphs and data can be found in the Supporting Information.

In an effort to identify any pathology correlates of the percentage of cells staining positive for FR-β, the FR-β positive fractions of both stromal and cancer cells was quantified by a trained pathologist using the multi-cancer TMA from Asterand. As summarized in Figure 5A, the fraction of cancer cells staining positive for FR-β did not correlate with the stage of the cancer, size of the primary tumor, involvement of lymph nodes or patient age. However, the percentage of positive cancer cells and stromal cells were both found to co-vary significantly with patient sex (*p*-value of 0.0001 and 0.00002, respectively) when sex specific cancers were removed from the analysis (breast and ovarian). Although the promoter for FR-α contains an estrogen regulatory element that leads to FR-α repression [31, 32], no such regulatory sequence has been reported for FR-β, leaving the explanation for this dramatic impact of gender on FR-β expression largely unanswered.

Unlike cancer cells, FR-β expression on stromal cells correlated positively with cancer stage and the closely related category of lymph node involvement (*p*-values of 0.0212 and 0.0293, respectively), even though none of the groups were significantly different from one another (Figure 5B). While the mechanistic underpinnings of this correlation still need to be explored, it has been established for some time that tumor-associated macrophages greatly increase the invasiveness and metastatic potential of tumors [33, 34, 35]. Because the tumor associated macrophages in this study were strongly FR-β positive, as reported previously for melanoma, pancreatic and head and neck cancers [9, 10, 23], the correlation with cancer stage and lymph node involvement suggests the intriguing possibility that FR-β might not only constitute a useful indicator of tumor metastatic potential, but may also serve as an attractive target for folate-mediated delivery of drugs for reprogramming the tumor's immune environment.

## CONCLUSION

Roughly a thousand tumor sections from 20 different cancers were stained with a new monoclonal antibody to human folate receptor β. Analysis of the stained sections indicate that FR-β is more prevalent in stromal cells than cancer cells and that expression on stromal cells is primarily found on tumor associated macrophages and macrophage-like cells. When correlations with other patient data were analyzed, FR-β expression was observed in a greater percentage of cells in females than males, and the percentage of positively staining stromal cells was correlated with the cancer's stage and lymph node involvement. Additionally, these data suggest that FR-β expression may be a useful indicator of a tumor's metastatic potential and that the receptor might be exploited for folate-mediated drug targeting to certain cancers and their associated anti-inflammatory macrophages.

## MATERIALS AND METHODS

### Materials

Horseradish peroxidase (HRP)-streptavidin and EZ-Link Sulfo-NHS-LC-Biotin were purchased from Thermo Scientific (Madison, WI). PeCy7-labeled streptavidin was obtained from eBioscience (San Diego, CA). RPMI1640 was purchased from Life Technologies (Grand Island, NY) and fetal bovine serum (FBS) was from Hyclone (Novato, CA). The cancer tissue microarrays that were examined in this study were: i) a hepatocellular carcinoma tissue microarray from Emory University (Atlanta, GA), ii) a thymoma tissue microarray from Indiana University-Purdue University Indianapolis (Indianapolis, IN), iii) a custom multi-tumor tissue microarray (TMA-00300) from Asterand (Detroit, MI), iv) a high density multiple organ tumor and normal tissue microarray (MC5003) from US Biomax (Rockville, MD), and v) a breast tumor microarray (ARY-HH0056), a brain glioma tumor microarray (ARY-HH0138) and an endometrial carcinoma progression tumor microarray (ARY-HH0211) from Folio Biosciences (Columbus, OH). Oregon green labeled folate was synthesized as previously described [36]. All other materials were purchased from VWR (Chicago, IL).

### Antibody

A human monoclonal anti-human FR-β antibody (m909) was generated against the extracellular domain (23~236 aa) of human FR-β and has been characterized previously in human samples [24]. m909 was labeled with EZ-Link Sulfo-NHS-LC-Biotin according to manufacturer's instructions.

### Flow cytometric analysis of the specificity of biotinylated-m909

CHO-β (human FR-β stably-transfected cell line generously provided by Dr. Manohar Ratnam), mock-infected parental CHO-K1 cells, and KB cells (human FR-α highly-expressing cell line) were trypsinized, washed in PBS, and resuspended in folate-deficient RPMI1640 plus 1% FBS. Aliquots of cells were incubated with serial concentrations of biotinylated-m909 (~0.7 mg/mL) or Oregon green labeled folate at 4°C for 1 hour. The cells then were washed once with ice-cold PBS and the antibody-labeled cells were stained with Streptavidin-PeCy7 (1:100 dilution) at 4°C for 1 hour. Cells then were washed once with ice-cold PBS, resuspended in PBS + 1% FBS and analyzed by flow cytometry using a FACSCalibur (Beckton Dickinson, San Jose, CA). Flow cytometry data were analyzed using Flowjo (TreeStar, Ashland, OR).

### Immunohistochemisty

All tumor microarrays had been previously fixed in formalin and embedded in paraffin by the provider. TMA samples were initially deparaffinized with 3 changes of xylene, rehydrated in a series of ethanol dilutions (100%, 95%, then 70% ethanol) and rinsed well in running distilled water. Slides then were placed in a preheated DAKO Target Retrieval buffer for 40 min then cooled in the buffer for 20 min followed by a 5 min rinse in running distilled water. After the heat inactivated epitope retrieval step, sections were incubated with 3% H_2_O_2_ in ethanol for 5 min to inactivate the endogenous peroxides, followed by a protein blocking step for 5 min. Slides were rinsed well with Tris-buffered saline containing 0.05% Tween 20 (TBST) wash buffer and incubated for 30 min at room temperature with biotinylated-m909 (~0.7 mg/mL). Sections then were rinsed with TBST wash buffer followed by the addition of HRP-streptavidin. After a 10 min incubation at room temperature, the slides were washed with TBST. Finally, slides were incubated in 3,3′-diaminobenzidine for 5 min at room temperature, counterstained with Modified Schmidts's Hematoxylin for 5 min and rinsed in tap water for 3 min. The samples then were dehydrated through graded alcohols, cleared in 3 changes of xylene and mounted with a permanent mounting media.

### Statistics

Differences between groups were determined by either a t-test (assuming equal variance and 2-tails for all samples) for data sets with only 2 groups or a 1-way ANOVA for data sets containing multiple groups. Correlation analyses were performed using a Spearman correlation analysis. Differences were considered statistically significant if the *p*-value was <0.05.

## SUPPLEMENTARY MATERIAL FIGURES


