# Supplementary material for: Assessment of folate receptor-β expression in human neoplastic tissues
Source: Oncotarget. 2015 Mar 30;6(16):14700–9. doi: 10.18632/oncotarget.3739 (PMC4546498; doi:10.18632/oncotarget.3739)
Supplement: Supplementary file 1 [file oncotarget-06-14700-s001.pdf]

Assessment of folate receptor-β expression in human neoplastic tissues

Supplementary Material

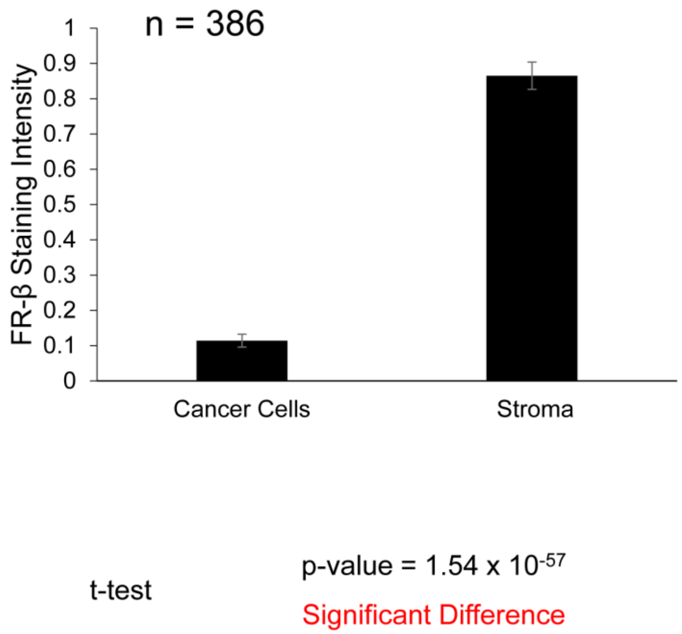

SI Figure 1: FR-β staining intensity of cancer and stromal cells. IHC was performed on a tissue microarray using the antibody m909. The staining intensity within the tumor and stroma were graded on a scale of 0 to 3 and the average value is plotted (error bars represent SEM). A t-test (assuming equal variance and 2-tailed) was used to determine if there were any statistically significant differences between cancer and stromal cells.

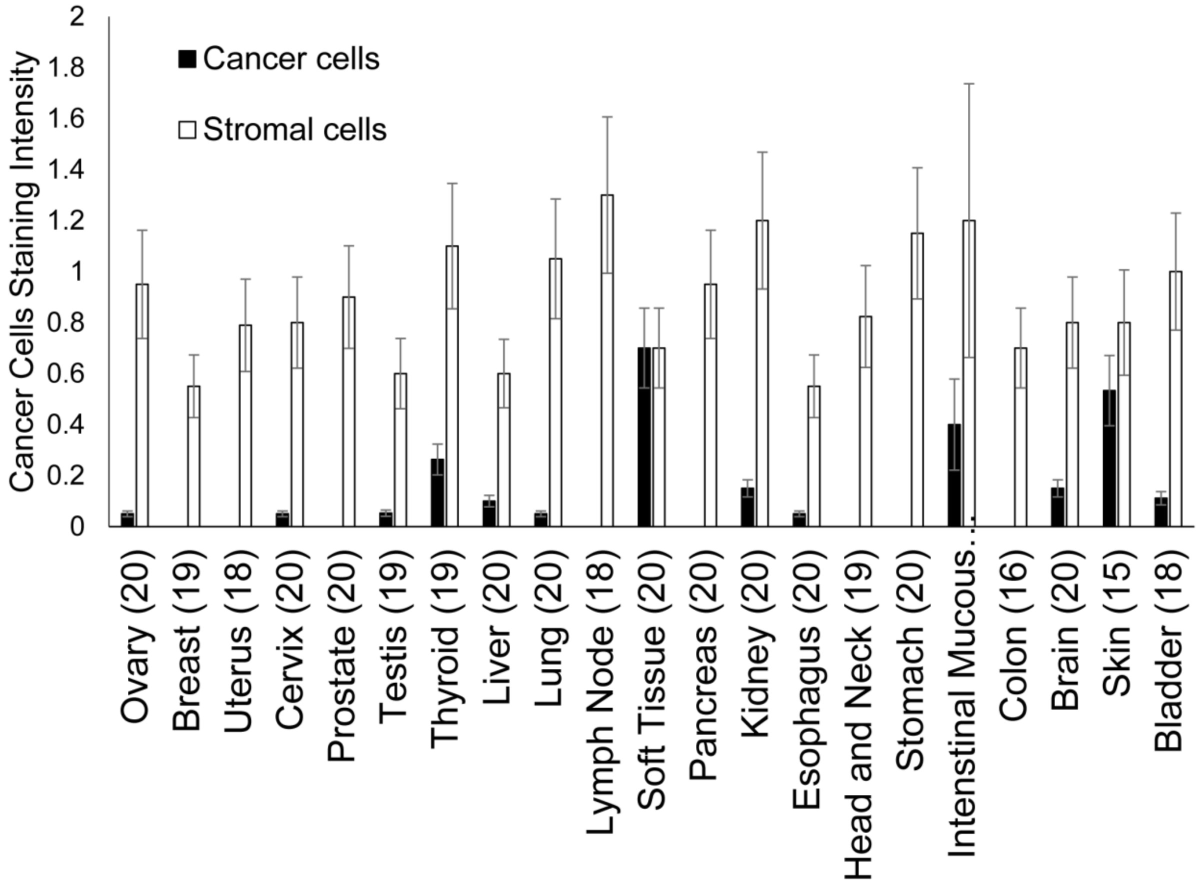

SI Figure 2: FR-β staining intensity in cancer and stromal cells. IHC was performed on a tissue microarray using the antibody m909. The staining intensity within the tumor and stroma were graded on a scale of 0 to 3. Average staining intensity is plotted for each cancer tissue type (error bars represent SEM) and the number of samples is shown in parenthesis.

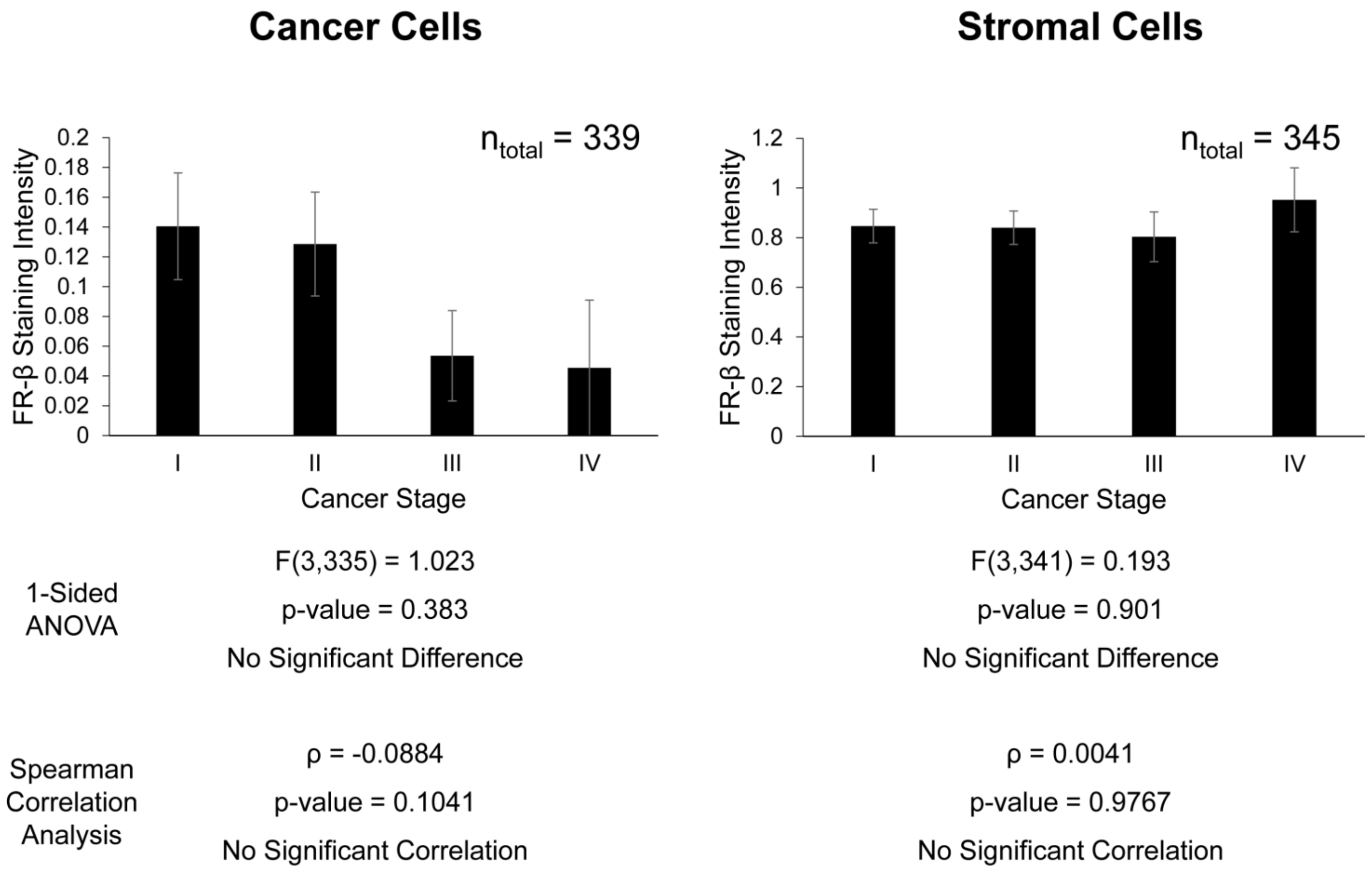

SI Figure 3: Correlation analysis of FR-β staining intensity and cancer stage. IHC was performed on a tissue microarray using the antibody m909. The staining intensity within the tumor and stroma were graded on a scale of 0 to 3 and the average is plotted (error bars represent SEM). A 1-sided ANOVA and Spearman correlation analysis were used to determine if there were any statistically significant differences between the individual groups and/or if there was a significant correlation between the intensity staining and the cancer stage**.**

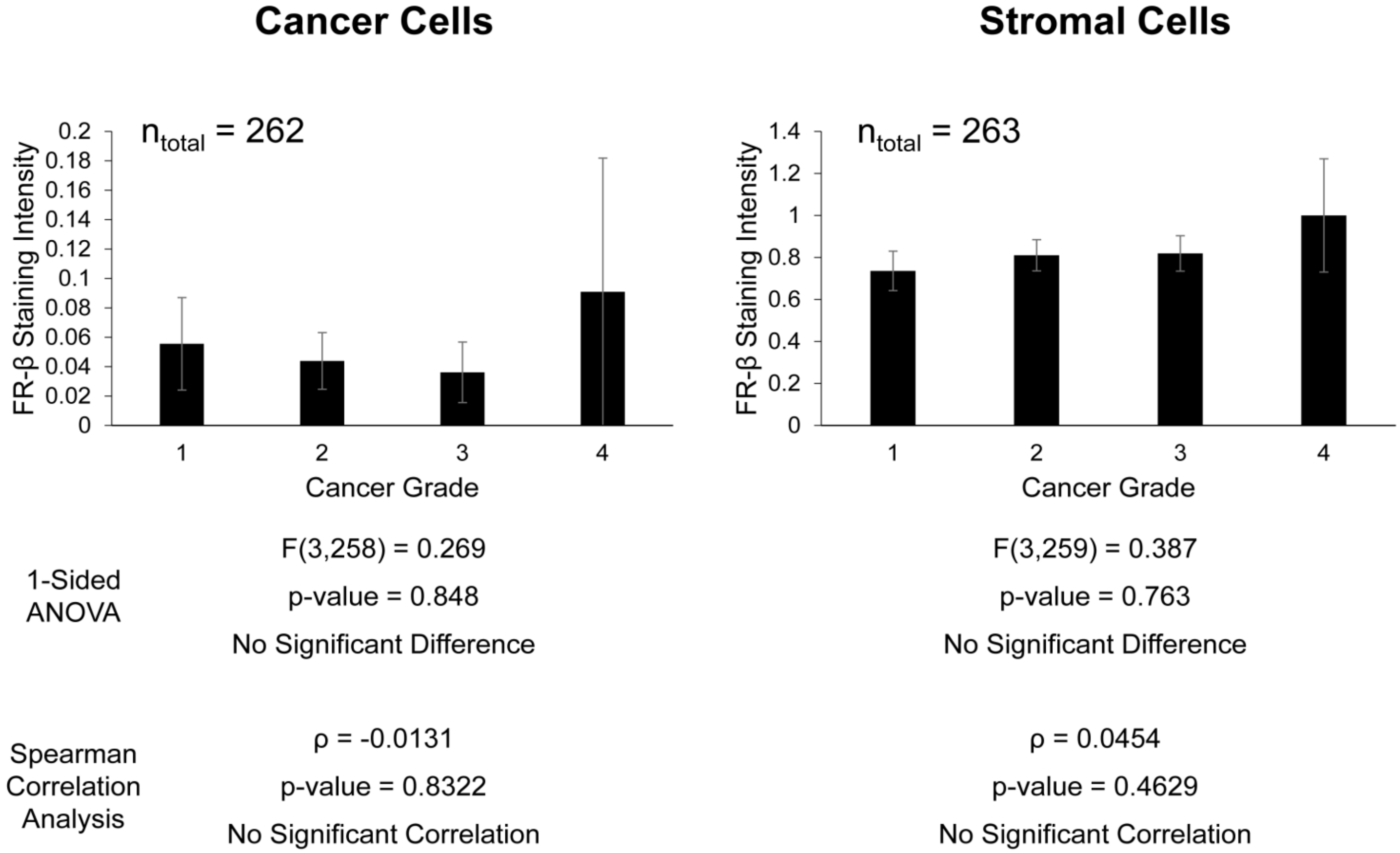

SI Figure 4: Correlation analysis of FR-β staining intensity and cancer grade. IHC was performed on a tissue microarray using the antibody m909. The staining intensity within the tumor and stroma were graded on a scale of 0 to 3 and the average is plotted (error bars represent SEM). A 1-sided ANOVA and Spearman correlation analysis were used to determine if there were any statistically significant differences between the individual groups and/or if there was a significant correlation between the intensity staining and the cancer grade.

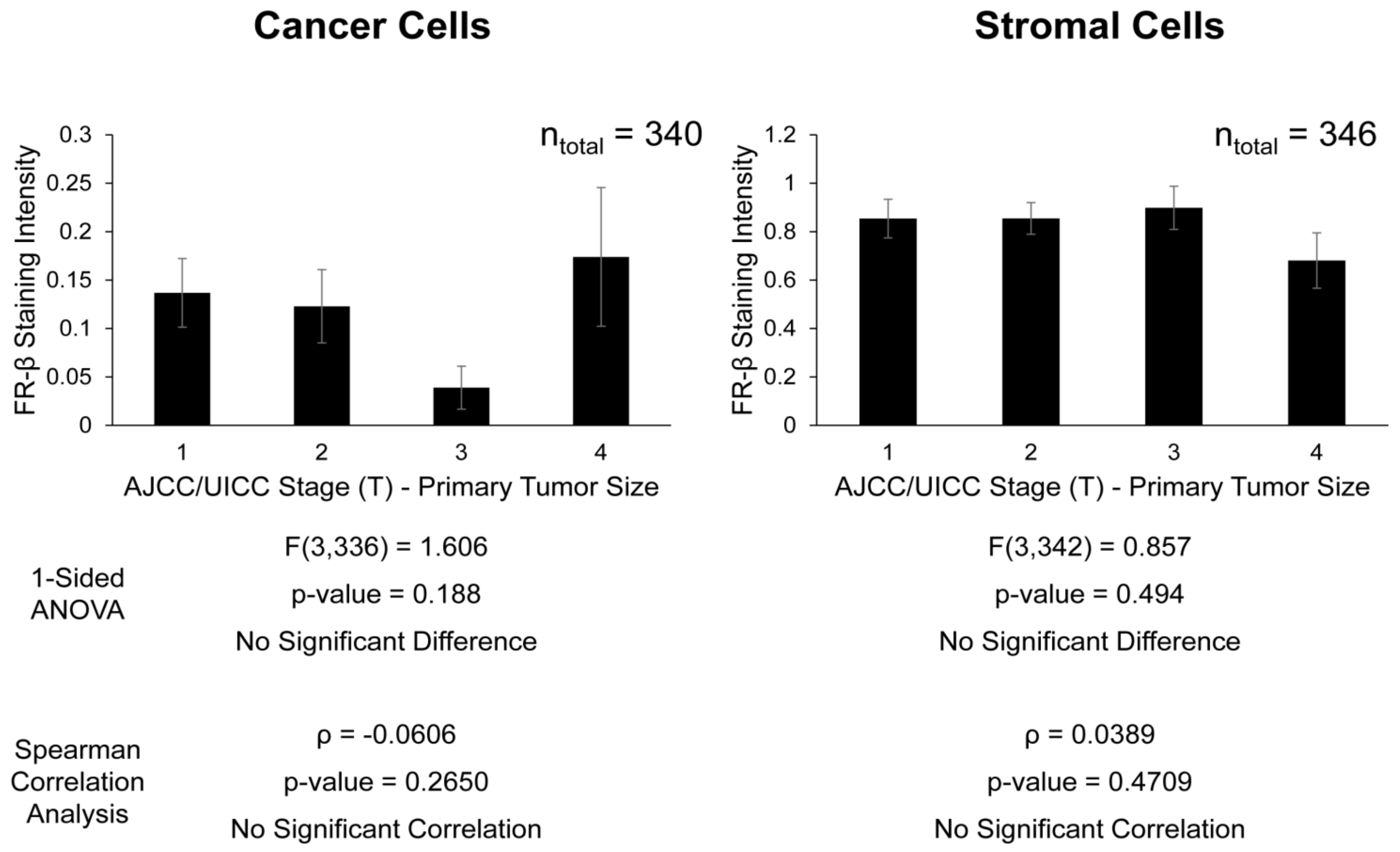

SI Figure 5: Correlation analysis of FR-β staining intensity and AJCC/UICC T value (size of primary tumor). IHC was performed on a tissue microarray using the antibody m909. The staining intensity within the tumor and stroma were graded on a scale of 0 to 3 and the average is plotted (error bars represent SEM). A 1-sided ANOVA and Spearman correlation analysis were used to determine if there were any statistically significant differences between the individual groups and/or if there was a significant correlation between the intensity staining and the primary tumor size.

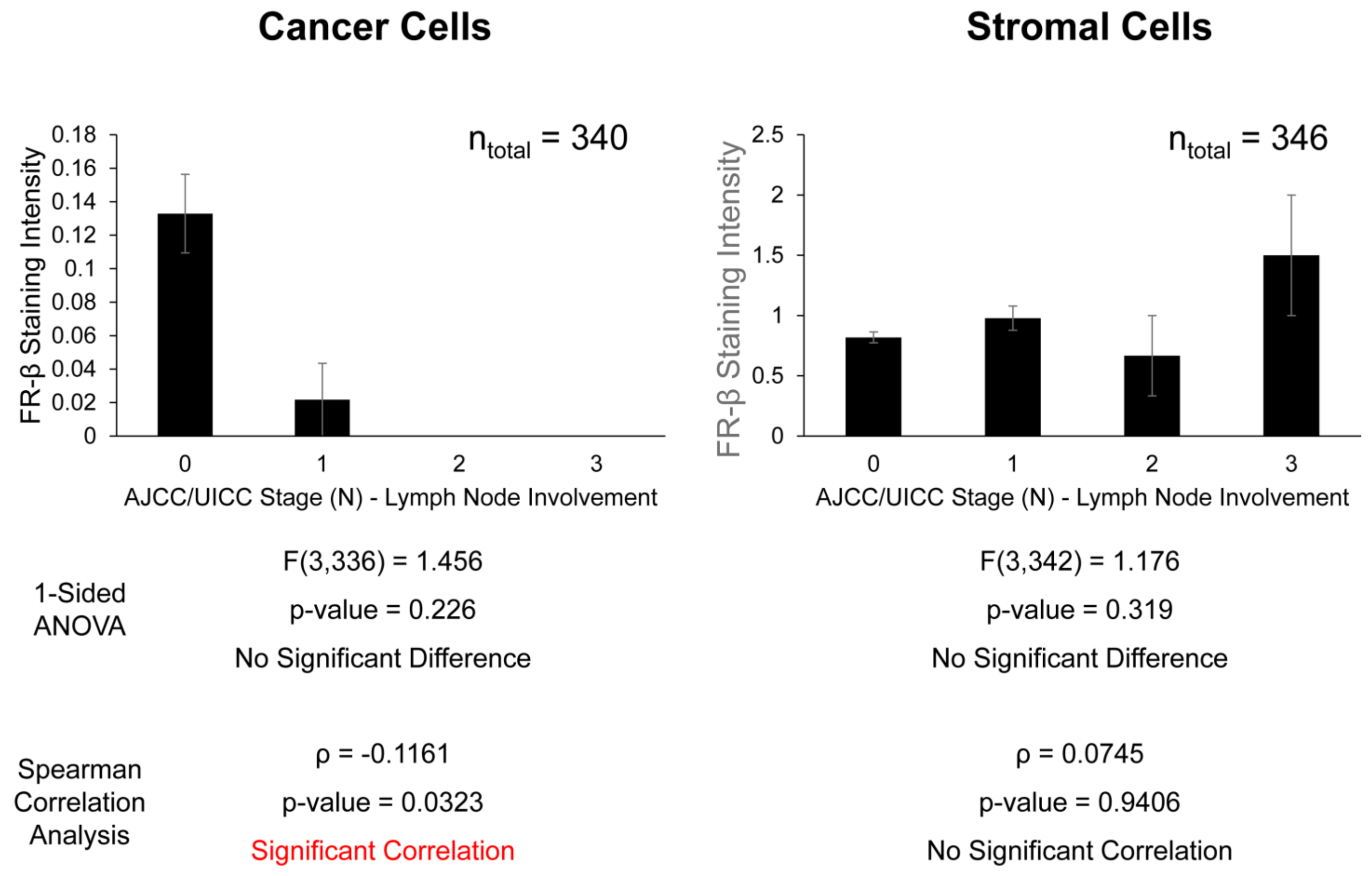

SI Figure 6: Correlation analysis of FR-β staining intensity and AJCC/UICC N value (level of lymph node involvement). IHC was performed on a tissue microarray using the antibody m909. The staining intensity within the tumor and stroma were graded on a scale of 0 to 3 and the average is plotted (error bars represent SEM). A 1-sided ANOVA and Spearman correlation analysis were used to determine if there were any statistically significant differences between the individual groups and/or if there was a significant correlation between the intensity staining and the lymph node involvement.

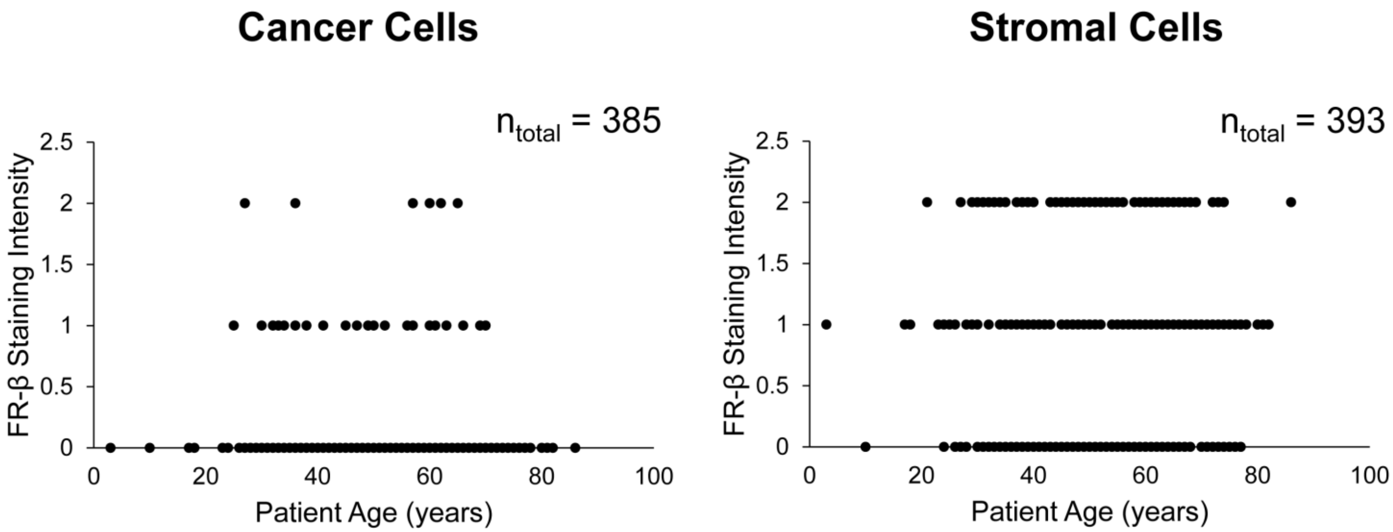

	Cancer Cells	Stromal Cells
1-Sided ANOVA	-<br>-<br>-	-<br>-<br>-
Spearman Correlation Analysis	ρ = -0.0608<br>p-value = 0.2339<br>No Significant Correlation	ρ = 0.0371<br>p-value = 0.4630<br>No Significant Correlation

SI Figure 7: Correlation analysis of FR-β staining intensity and patient age at cancer excision. IHC was performed on a tissue microarray using the antibody m909. A Spearman correlation analysis were used to determine if there was a significant correlation between the intensity staining and the patient's age.

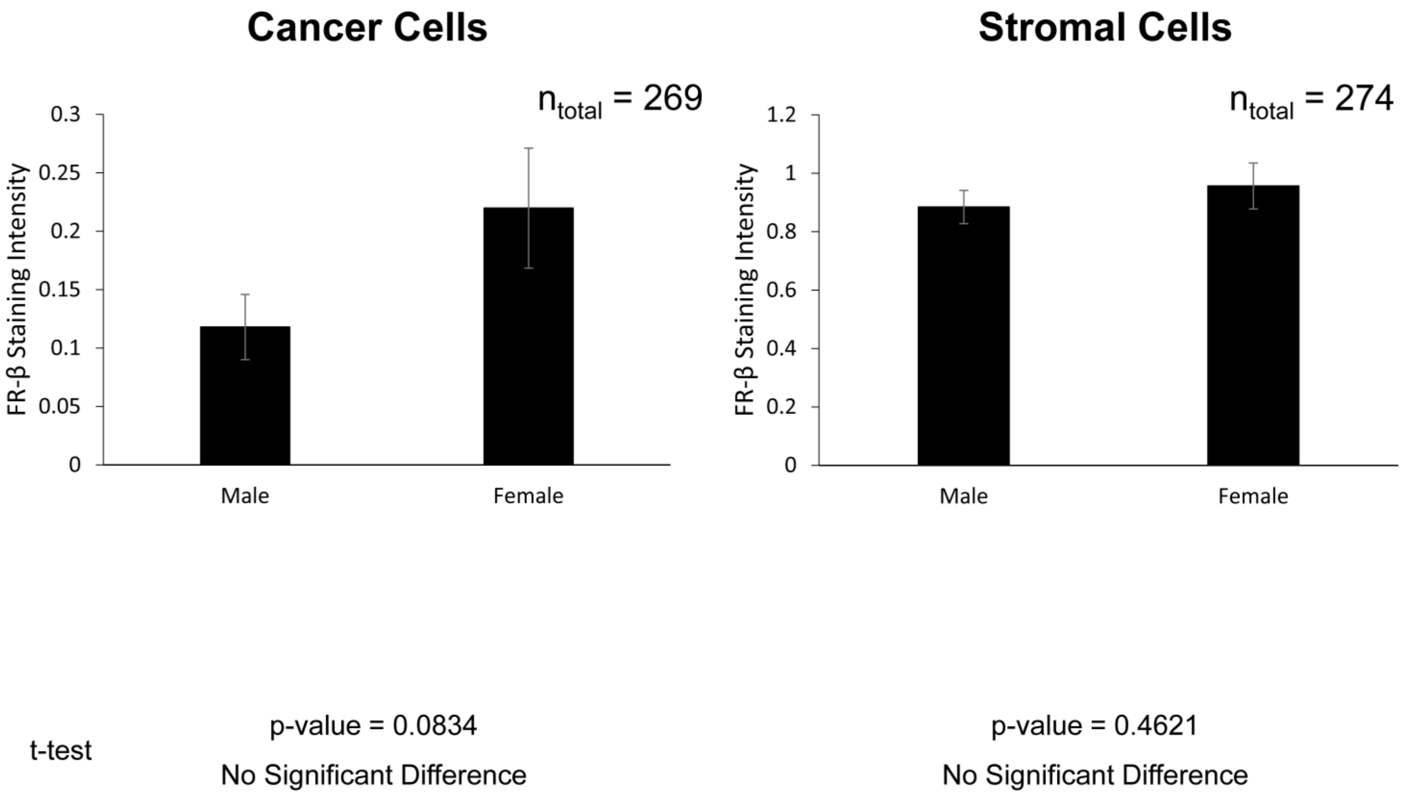

SI Figure 8: Correlation analysis of FR-β staining intensity and patient sex. IHC was performed on a tissue microarray using the antibody m909. The staining intensity within the tumor and stroma were graded on a scale of 0 to 3 and the average is plotted (error bars represent SEM). A t-test (assuming equal variance and 2-tailed) was used to determine if there were any statistically significant differences in staining intensity between males and females.

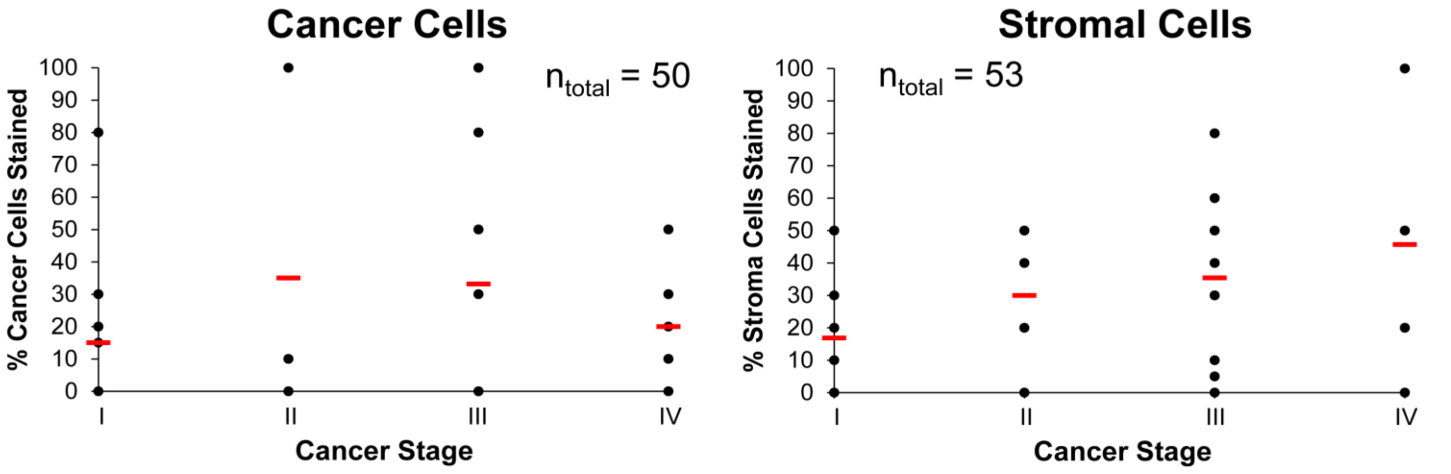

	Cancer Cells	Stromal Cells
1-Sided ANOVA	$F(3,46) = 1.021$<br>p-value = 0.392<br>No Significant Difference	$F(3,49) = 2.436$<br>p-value = 0.076<br>No Significant Difference
Spearman Correlation Analysis	$\rho = 0.2030$<br>p-value = 0.1575<br>No Significant Correlation	$\rho = 0.3159$<br>p-value = 0.0212<br>Significant Correlation

SI Figure 9: Correlation analysis of FR-β % cells staining positive and cancer stage. IHC was performed on a tissue microarray using the antibody m909. The approximate percentage of positively staining cells within the tumor and stroma were determined (red bars represent population mean). A 1-sided ANOVA and Spearman correlation analysis were used to determine if there were any statistically significant differences between the individual groups and/or if there was a significant correlation between the percentage of positive staining cells and the stage of the cancer.

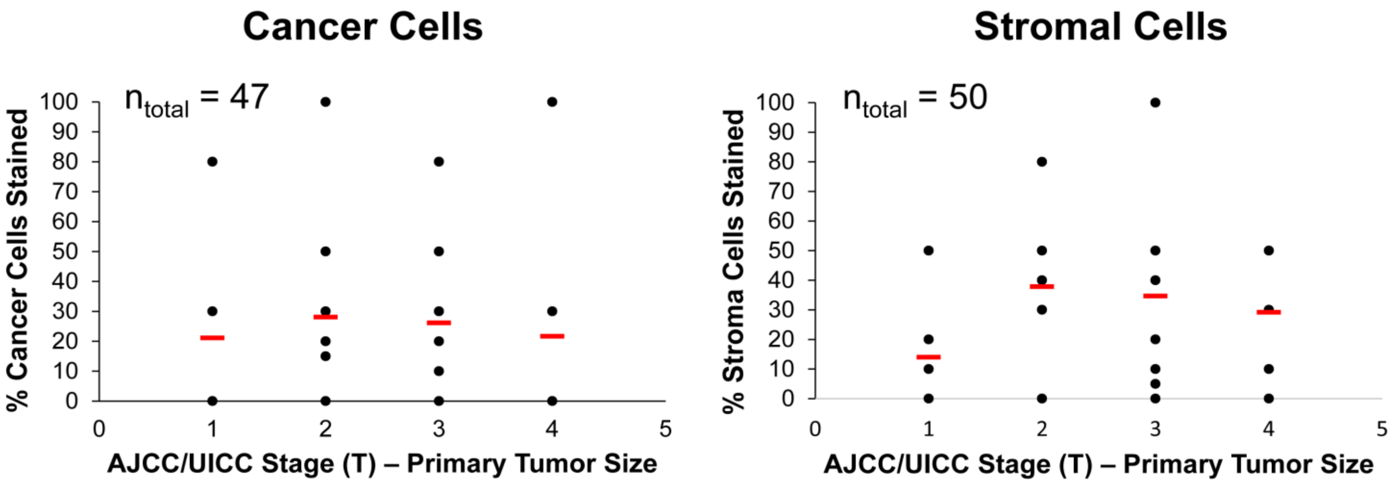

	Cancer Cells	Stromal Cells
1-Sided ANOVA	$F(3,43) = 0.111$<br>P-value = 0.953<br>No Significant Difference	$F(3,46) = 1.694$<br>p-value = 0.181<br>No Significant Difference
Spearman Correlation Analysis	$\rho = -0.0036$<br>p-value = 0.9808<br>No Significant Correlation	$\rho = 0.1382$<br>p-value = 0.3386<br>No Significant Correlation

SI Figure 10: Correlation analysis of FR-β % cells staining positive and AJCC/UICC T value (size of primary tumor). IHC was performed on a tissue microarray using the antibody m909. The approximate percentage of positively staining cells within the tumor and stroma were determined (red bars represent population mean). A 1-sided ANOVA and Spearman correlation analysis were used to determine if there were any statistically significant differences between the individual groups and/or if there was a significant correlation between the percentage of positive staining cells and the size of the primary tumor.

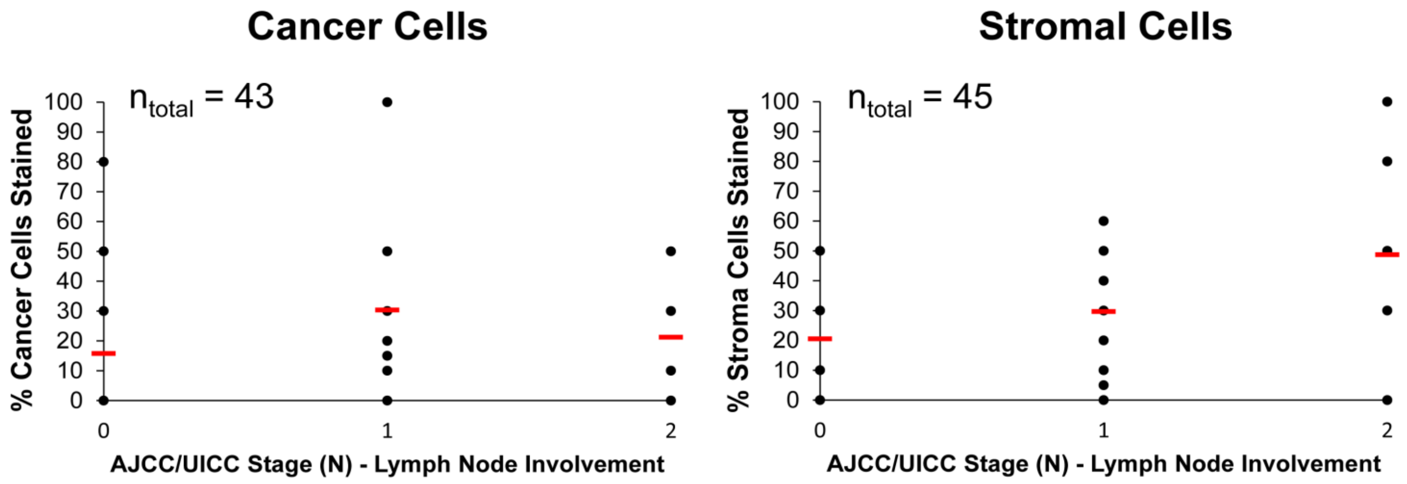

	Cancer Cells	Stromal Cells
1-Sided ANOVA	$F(2,38) = 0.862$<br>P-value = 0.430<br>No Significant Difference	$F(2,40) = 3.508$<br>p-value = 0.039<br>Significant Difference
Spearman Correlation Analysis	$\rho = 0.1905$<br>p-value = 0.2212<br>No Significant Correlation	$\rho = 0.3250$<br>p-value = 0.0293<br>Significant Correlation

SI Figure 11: Correlation analysis of FR-β % cells staining positive and AJCC/UICC N value (level of lymph node involvement). IHC was performed on a tissue microarray using the antibody m909. The approximate percentage of positively staining cells within the tumor and stroma were determined (red bars represent population mean). A 1-sided ANOVA and Spearman correlation analysis were used to determine if there were any statistically significant differences between the individual groups and/or if there was a significant correlation between the percentage of positive staining cells and the level of lymph node involvement.

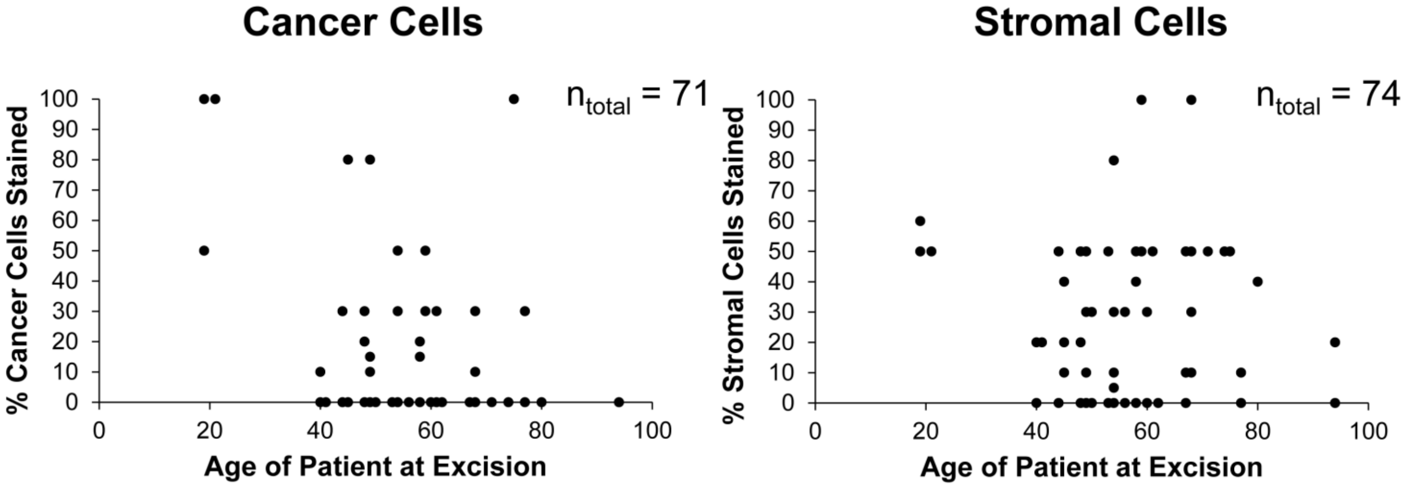

	Cancer Cells	Stromal Cells
1-Sided ANOVA	- - -	- - -
Spearman Correlatio n Analysis	ρ = -0.2337 p-value = 0.0501 No Significant Correlation	ρ = -0.0285 p-value = 0.8096 No Significant Correlation

SI Figure 12: Correlation analysis of FR-β % cells staining positive and patient age at tumor excision. IHC was performed on a tissue microarray using the antibody m909. The approximate percentage of positively staining cells within the tumor and stroma were determined. A Spearman correlation analysis was used to determine if there was a significant correlation between the percentage of positive staining cells and the patient age at tumor excision.

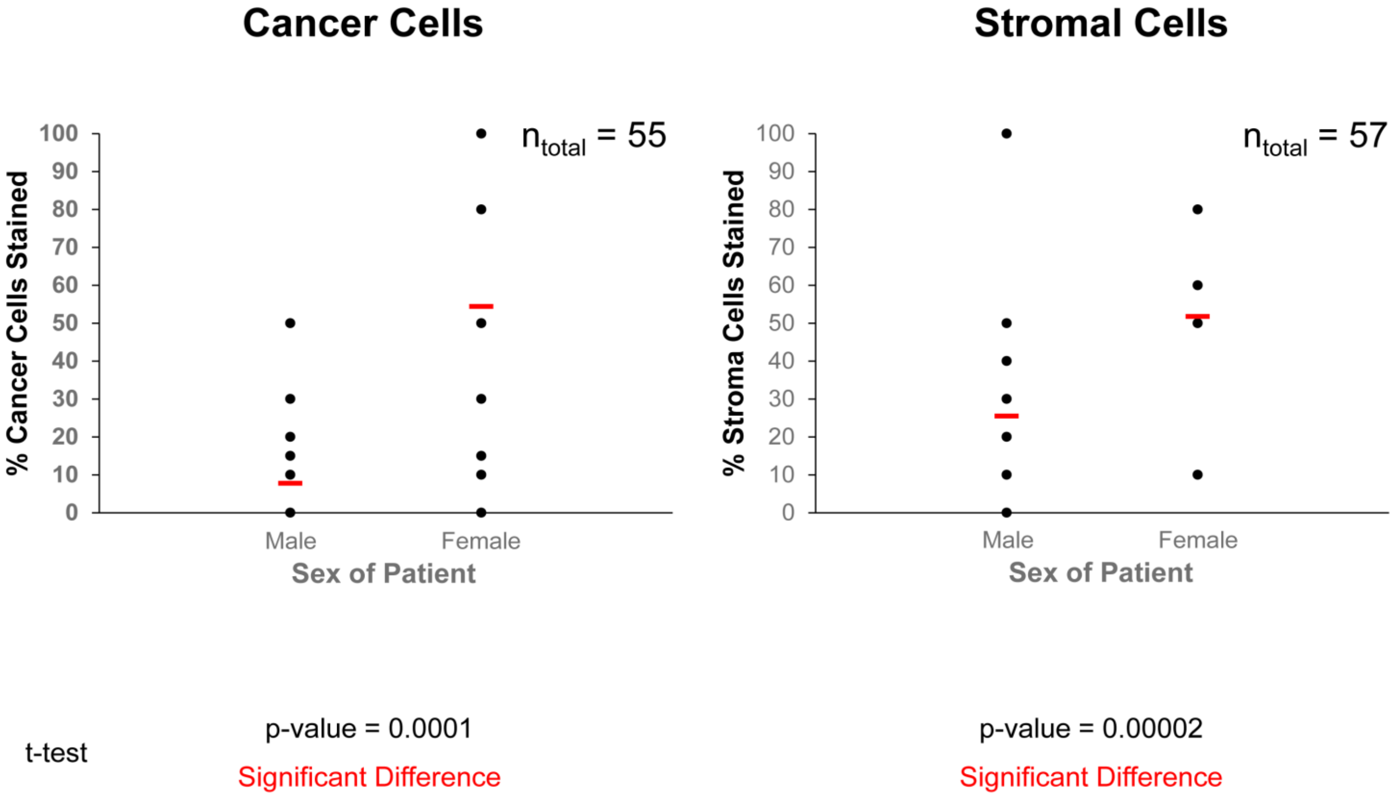

SI Figure 13: Correlation analysis of FR-β % cells staining positive and patient sex. IHC was performed on a tissue microarray using the antibody m909. The approximate percentage of positively staining cells within the tumor and stroma were determined (red bars represent population mean). A t-test (assuming equal variance and 2-tailed) was used to determine if there were any statistically significant differences between males and females.
